# Rabies in Our Neighbourhood: Preparedness for an Emerging Infectious Disease

**DOI:** 10.3390/pathogens10030375

**Published:** 2021-03-20

**Authors:** Michael P. Ward, Victoria J. Brookes

**Affiliations:** 1 Sydney School of Veterinary Science, The University of Sydney, Camden, NSW 2570, Australia; 2 School of Animal and Veterinary Sciences, Faculty of Science, Charles Sturt University, Wagga Wagga, NSW 2678, Australia; vbrookes@csu.edu.au; 3 Graham Centre for Agricultural Innovation (NSW Department of Primary Industries and Charles Sturt University), Wagga Wagga, NSW 2678, Australia

**Keywords:** rabies, lyssavirus, dog, environmental scanning, risk assessment, disease spread modelling, surveillance, response, policy, Australia

## Abstract

Emerging infectious disease (EID) events have the potential to cause devastating impacts on human, animal and environmental health. A range of tools exist which can be applied to address EID event detection, preparedness and response. Here we use a case study of rabies in Southeast Asia and Oceania to illustrate, via nearly a decade of research activities, how such tools can be systematically integrated into a framework for EID preparedness. During the past three decades, canine rabies has spread to previously free areas of Southeast Asia, threatening the rabies-free status of countries such as Timor Leste, Papua New Guinea and Australia. The program of research to address rabies preparedness in the Oceanic region has included scanning and surveillance to define the emerging nature of canine rabies within the Southeast Asia region; field studies to collect information on potential reservoir species, their distribution and behaviour; participatory and sociological studies to identify priorities for disease response; and targeted risk assessment and disease modelling studies. Lessons learnt include the need to develop methods to collect data in remote regions, and the need to continuously evaluate and update requirements for preparedness in response to evolving drivers of emerging infectious disease.

## 1. Introduction

Priorities for epidemiologic investigation of disease are dynamic: they change continuously on global and local scales, as well as over the short and long term. In the 1960s, following the advent of antimicrobials and vaccination, epidemiologic focus shifted towards chronic, non-infectious disease; further advancement of the control of infectious disease was considered more likely to be delivered through laboratory-based research rather than epidemiology [1]. However, subsequent emergence and re-emergence of animal and human infectious diseases demonstrated that disease circumstances—and thus priorities—evolve. Epidemiologic investigation informed responses to major animal and human infectious diseases events such as bovine spongiform encephalopathy (BSE), new variant Creutzfeld–Jakob disease (nvCJD), acquired immunodeficiency syndrome (AIDS) and methicillin-resistant *Staphylococcus aureus* (MRSA) in the 1980s and 1990s [2,3,4,5]. By the early 2000s, it was clear that the incidence of emerging infectious disease (EID) had increased during the previous decades [6] and that EIDs would likely remain a focus of epidemiologic research.

As expected, the impacts of EIDs continue in the 21st century. Diseases newly emerge (for example, those caused by novel coronaviruses such as MERS-CoV, SARS-CoV and SARS-CoV-2 [7]), re-emerge as large outbreaks (for example, Ebola and Zika [8,9]), or re-emerge despite ongoing control measures (for example, canine rabies [10]). The broad circumstances of EIDs are well described; some species (such as bats) appear more likely to be reservoir hosts for potential pathogens, and interfaces that provide opportunity for pathogen transmission are more likely in regions experiencing the impacts of climate change and anthropogenic activities (such as deforestation, urbanization and intensive livestock farming) [6,11,12]. Predicted disease emergence can be further refined to regions with limited human health capacity and opportunity for air travel that can disseminate disease to urban centers globally [13]. However, prediction of the precise circumstances of emergence is not possible; therefore, we rely on a range of epidemiologic methods and tools to inform prevention strategies, detection and response.

A systematic approach that integrates epidemiologic methods has previously been described [14]. The approach gathers information inputs—generated via tools such as environmental scanning, horizon scanning and surveillance—in a cycle in which information is collected and assessed, threats are prioritised, and risk assessment and disease simulation modelling are used to generate key information for policy makers. By systematically integrating these components, the goal is not only to reduce the probability of an EID event and to mitigate its impact, should it occur, but also to identify gaps in current preparedness strategies and provide opportunities to re-assess evolving drivers of disease emergence. In some circumstances, such changes would require re-assessment and subsequent re-alignment or updating of preparedness strategies to continue to meet the needs of a region. Given the interconnected nature of the drivers of EID emergence, their social, economic and political underpinnings, and their cross-sectoral impacts, this approach provides opportunity for a One Health approach for EID preparedness and response.

**Figure 1:**
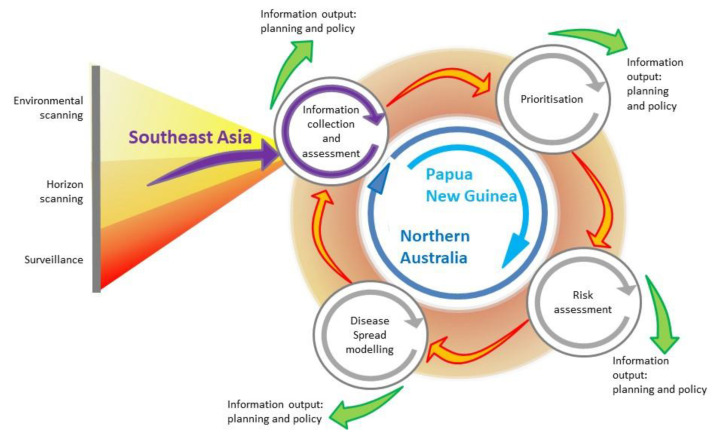
Emerging infectious disease preparedness framework, integrating surveillance, horizon and environmental scanning, prioritisation, risk assessment and disease modelling (modified from [14]). The framework illustrates information collection and assessment from Southeast Asia, and the activities undertaken in Papua New Guinea and northern Australia, in a program of research for rabies preparedness in the Oceanic region.

In this review, we evaluate a program of research conducted between 2012 and 2019 in response to changes in the distribution of rabies in Southeast Asia, in the context of this systematic, integrated approach to disease preparedness. Although rabies is endemic in many parts of the world, it has recently re-emerged in historically or previously free regions of Indonesia and Malaysia. The program aimed to develop strategies for rabies preparedness and response in Australia given the perceived increase in risk of a rabies incursion in the Oceanic region. Figure 1 illustrates the framework and provides an overview of how it was implemented during the program. In terms of using a framework for integration of epidemiologic methods and tools, we assess the extent to which implementation of this framework was feasible in this context. We describe the barriers and limitations, and whether outcomes for preparedness and response were enhanced. We also assess whether a systematic approach using such a framework is useful to identify gaps, both in underlying knowledge required for rabies research in the region, as well as gaps in outcomes from the research. Lastly, we make recommendations for further epidemiologic research for rabies in the Oceanic and Southeast Asian regions, and about how the framework could be integrated in a broader One Health agenda, particularly in the context of enhancing policy.

## 2. The Nature of Rabies: A Summary

Rabies is one of humanity’s oldest neglected infectious diseases [15]. Rabies virus (RABV) is a member of the genus *Lyssavirus*; it causes an acute, progressive neurological disease in susceptible species. The genus *Lyssavirus* contains more than 16 species, generally maintained in wildlife (and often bats). RABV is the most widespread *Lyssavirus* and although RABV can infect all mammals, the most common maintenance hosts are canids.

It is likely that Eurasia was the historical source for the spread of canine rabies to other regions, centuries ago, via trade and colonization [15]. Rabies is globally distributed, estimated to cause approximately 59,000 human deaths worldwide and cost approximately 8.6 billion USD annually [16]. Global Alliance on Rabies Control (GARC) together with partners (World Health Organisation, WHO; Food and Agriculture Organisation of the United Nations, FAO; World Organisation for Animal Health, OIE) launched the framework for the elimination of dog-mediated human rabies in 2015, and the Zeroby30 Global Strategic Plan in 2018 to end human deaths from dog-mediated rabies by 2030.

The most common source of human RABV infection are domestic dogs, responsible for >95% of human cases [17]. Rabies has one of the highest case-fatality rates of infectious diseases, with virtually all untreated cases dying. The most common route of RABV transmission is an animal bite directly injecting RABV contaminated saliva into a susceptible individual. In dogs, it has been estimated that approximately 50% of exposures cause infection [18]. The incubation period is highly variable, dependent on viral load, site of viral injection (closer to the head tends to have shorter incubation period), viral strain and immunological status of the dog [19]. Reconstructed case studies have been used to estimate incubation periods in dogs in Tanzania (22 days [18]) and Tokyo (27 days [20]).

Clinical signs of rabies in animals can only be used for presumptive diagnosis because the different forms of clinical rabies (furious or paralytic) have signs that are similar to other diseases, and vary between individuals. Definitive rabies diagnosis is via laboratory tests to identify rabies viral particles in tissue samples from suspect animals. The gold standard test recommended by the WHO and the OIE is the direct fluorescent antibody (DFA) test [17,21], in which a fluorescent-tagged anti-rabies antibody conjugate binds with rabies viral antigens if present in tissue samples (usually brain). Used by a skilled technician, the DFA test is highly sensitive and specific (96–99% [21]). Comparable tests include the direct rapid immunohistochemistry test (dRIT), and reverse transcriptase polymerase chain reaction (RT-PCR) assays. Although these diagnostic tests are accurate, their application in rural areas and lower-income countries can be challenging.

Interventions focused on rabies elimination in dogs are the key to reducing human rabies incidence and recurrent costs [22,23,24]. Although mass vaccination of dogs with a minimum 70% coverage of the population is an expensive program in resource-limited countries, relying on international agencies for financial support [25,26], the unit cost per dog vaccination is lower than human post-exposure prophylaxis (PEP) cost [27] or programs to kill dogs [28]. 

The rabies situation in Asia is characterised by high human mortality: it is estimated that more than 35,000 people in Asia die each year from rabies, which represents 60% of the global burden [16]. Only a few countries in Asia (Japan, Singapore, Malaysia, Taiwan, Hong Kong, South Korea) have eliminated canine rabies through mass dog vaccination and stray dog population control programs, for example elimination of canine rabies in West Malaysia in the 1950s [29,30,31,32,33].

Currently, rabies is endemic in all Southeast Asian countries, except Singapore, Timor-Leste and Brunei Darussalam [34]. All of Oceania (including West Papua and Papua provinces, Indonesia) is canine rabies free. The annual human death burden in Southeast Asia has been estimated to be 5,823 (10% of the global burden [16]).

## 3. The Long-Term Risk of RABV to Australia: Foresighting, Environmental and Horizon Scanning, and Surveillance

Historically, Australia has remained canine rabies free. RABV infection has never been documented in the native fauna of Australia. An incursion of RABV was suspected around Hobart (in the island state of Tasmania) in the period 1866–1867 [35,36,37]. Up to five dogs and three pigs were reportedly affected, and five people were bitten (one death) by dogs. It was assumed that RABV was introduced via an infected, incubating dog on a ship, probably in August 1866. Seizure and destruction of stray dogs likely brought the outbreak to an end by February 1867 [37]. Two confirmed cases of rabies in humans in Australia in the 1990s have been reported, but both were contracted outside of Australia [38,39].

Consequently, due to strong quarantine regulations on the importation of susceptible species, the risk of RABV to Australia was largely discounted. However, in the 1980s there was increasing recognition of disease threats to northern Australia [40]. Dingoes, domestic dogs and their hybrids in northern Australia were recognised as potential reservoir populations in the event of an RABV incursion [41]. Forman [42] highlighted the potential of dogs to become infected and maintain both a sylvatic (involving red foxes which were introduced to Australia in 1871, but are not present in northern Australia) and urban rabies cycle in Australia. The susceptibilities of possums and other Australian native fauna, including carnivorous quolls (*Dasyurus* spp.) is unknown, but due to presumed low abundance and limited distributions, native carnivorous mammals have not been considered a high risk for RABV transmission in Australia [43]. Rabies was included in Australia’s animal disease preparedness plans (AUSVETPLAN) to address the incursion of exotic disease [44], the first edition of which was published in 1991. In addition, an exercise to test fox rabies preparedness and contingency planning was conducted in 1990 [45].

The recognition that northern Australia faced disease threats from both regulated and unregulated (associated with illegal activities) pathways—which were expected to increase in activity as this region was developed in response to economic drivers—and that a large, susceptible and potential reservoir species existed in this region, thus increasing the risk of RABV to Australia is an example of using information from environmental and horizon scanning (Figure 1). These are broad methods used to detect long-term environmental trends (in the broadest sense) and health threats, respectively. They complement traditional surveillance methods which are usually more narrowly focused in terms of geographical and temporal scope. In the context of preparedness for EIDs, early detection is critical and identifying the changing threats to health that drive EIDs is the starting point of the strategic planning process.

Environmental scanning to enhance EID preparedness is usually developed on an *ad hoc* basis to meet current and anticipated needs [46]. It collects information with little or no disease specificity, very broad geographical and disciplinary scope, and usually a high degree of uncertainty. It depends on collection of information over relatively long time periods to address the issue of uncertainty. Selecting the relevant information and assessing its quality is challenging, and a potential limitation of environmental scanning [47]. Information sources that are scanned in this process include a wide range of literature (peer-reviewed and grey literature, government reports and web-based information; for example blogs, list servers and other information networks) and informal data sources such as public opinion polls and media reports, as well as expert opinion elicitation and industry workshops. Horizon scanning (often used synonymously with environmental scanning) is used to describe information collection about adverse health events.

Information collected from scanning can be used in foresight activities such as scenario planning, causal layered analysis, and backcasting [48,49,50]. Predicted scenarios are not disease specific, but the process of environmental scanning has been applied to infectious disease emergence to enhance preparedness [49,51]. Once plausible future scenarios are identified, then the focus becomes assessing requirements to achieve or mitigate the chances of reaching those scenarios, such as policy changes or technology development.

**Figure 2:**
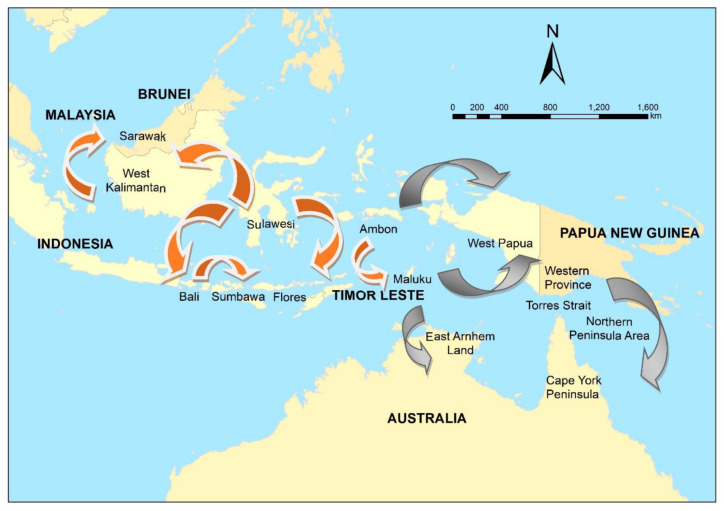
The spread of canine rabies in Indonesia and Malaysia, 1997–2019 (orange), and potential risk pathways (grey) to Papua New Guinea and Australia.

In 1997, canine rabies spread to Flores Island, Indonesia [52], then Ambon Island in 2003 [52], and Bali in 2008 [53,54]. Insufficient vaccination, and an initial focus on dog elimination, resulted in failure to eliminate rabies in Bali [53,55]. The incursion of rabies in Flores, officially confirmed in April 1998, was traced back to three suspected rabid dogs brought from Buton Island, Sulawesi by a fisherman in September 1997 [56]. Likewise, the Bali incursion in 2008 was thought to have been initiated when a fisherman landed on the Bukit peninsula in the far south of the island with a dog that was incubating the virus (Putra et al. in [57]) (Figure 2).

Thus, a link between the movement of dogs via inter-island fishing and rabies spread in eastern Indonesia was known. Within an environmental scanning framework, changes in fishing patterns—for example, in response to changing markets and economic drivers—would be expected to have an impact on the risk of rabies incursions. It is of note that traditional fishing by the Buginese and Makasarese people of southern Sulawesi has occurred in this region for centuries; prior to the British colonization of northern Australia in the late 19th century, fishing on the Arnhem Land coast of northern Australia occurred, as well as trade with the local Aboriginal people. 

Although formal foresighting activities were not conducted, this emergence of rabies in previously free regions in eastern Indonesia prompted a re-evaluation of the risk to the Oceanic region [58]. The risk of a rabies incursion in northern Australia was assessed qualitatively using expert opinion in 2012 [59] based on the probability of entry (proximity to Indonesia and fishing, trade, and cultural travel routes) and potential impacts along the coastline (presence of communities in which domestic dogs free-roamed). Sparkes et al. [60] summarised potential incursion scenarios in two high priority regions—East Arnhem Land in the Northern Territory, and the Northern Peninsula Area (NPA) in Far North Queensland (potentially via currently rabies-free Papua New Guinea (PNG) and the Torres Strait)(Figure 2). Entry pathways included the illegal importation of rabid dogs travelling on boats engaged in Australian—South East Asian cross-cultural traditions, unauthorised fishing vessels, and itinerant yachts (Figure 2).

The rabies research program described below was then instigated to investigate the risk of an incursion to Australia and neighbouring PNG, and the response and control strategies that would be required given the potential impact of rabies in Australia. The program integrated a variety of methods from the field of prioritisation, risk assessment and disease modelling (Figure 1).

## 4. Prioritisation and Risk Assessment: The Current Risk of RABV to Australia

In the first stage of the program, prioritisation of routes of incursion and detailed risk analysis of these routes were undertaken. Given the high-risk areas for a rabies incursion along the northern Australian coastline and the possibility of an incursion to PNG (and hence to northern Australia), these areas were prioritised.

Prioritisation uses methods borrowed from the field of decision analysis, that range from qualitative ranking systems [59] to quantitative multi-criteria decision analysis and discrete choice experiments [61,62]. A prioritisation exercise comprises a group of entities or activities that need to be ranked, and a group of criteria or attributes on which the ranking is based [63]. Prioritisation can have many purposes in the context of EIDs, including identification of potentially high impact EIDs to direct surveillance [64], and prioritising resource allocation for a particular disease such as rabies [65]. The geographic context of a prioritisation exercise in usually broad (for example, regional or country-wide) and thus, the population to which the prioritisation applies is usually large.

Risk analysis also comprises qualitative and quantitative methods. It is used in contexts such as import risk analysis to support trade of animals and their products, and food safety. In contrast to prioritisation, the scope is usually narrower and more specific, i.e., a single risk pathway is considered for a defined disease risk. Whilst entry and exposure pathways are included, evaluation of the ongoing impact to a population is addressed by other methods such as scenario or disease modelling. For example, in a study of the impact of West Nile virus in Australia, risk analysis was used to investigate the probability of entry and exposure, and scenario modelling based on the expected spatiotemporal spread was used to investigate equine and human population impact [66]. Therefore, unless other methods are included, the geographic scope and the population at-risk of exposure in risk analyses is relatively focused compared to prioritisation. Ward and Hernandez-Jover [67] produced a generic risk assessment tool for the entry of rabies, to support surveillance activities, and examples of implemented rabies risk assessments include investigation of the impact of a change to the United Kingdom (UK) Pet Travel Scheme (PETS), and assessment of the entry of rabies into Taiwan [68]. In the studies in the UK and Taiwan, rabies incursion risk was largely driven by non-compliance with border regulations, demonstrating how risk analyses can be used to highlight useful disease control points. 

In the rabies program we present here, Cape York Peninsula, Queensland was already identified as high priority for a rabies incursion [59], and to further inform surveillance strategies, the risk of a rabies incursion to this region was investigated in greater detail using risk analysis [69]. Structured interviews were conducted with key informants in the Cape York Peninsula, which confirmed that illegal boats from Indonesia were considered a high-risk pathway. A quantitative risk assessment was then conducted to investigate the probability of entry of rabies into north-west Cape York Peninsula via an infected dog on this pathway, followed by the probability exposure of a dog resident in a remote community. This identified that although the probability of exposure of a resident dog to rabies from the particular pathway was extremely low, the estimates were influenced by the prevalence of rabies in dogs at the origin of the route in Indonesia.

In contrast to northern Australia, prioritisation of regions, modes or routes of entry for rabies into PNG had not been conducted. Initially, modes of entry were prioritised in a qualitative exercise in which the likely number of dogs (not necessarily rabies infected) were estimated given the volume of types of traffic, number of people and purpose or reason for travel [70]. Logging, fishing and border crossings on foot (from Papua Province, Indonesia) were identified as high priority modes of incursion, and further risk assessment on these routes to identify high priority regions [71]. Innovative methods were used, such as elicitation of expert-opinion mainly via online engagement, and use of a generic risk assessment pathway so that prioritisation by comparison of the risk of incursion via any mode in any region was feasible. The investigation was extensive, using 8 questionnaires and parameterisation of >220 empirical distributions. The highest risk modes of incursion were border crossings on foot, and the highest risk-region was Western Province. The volume of traffic on each route was the most influential driver of risk. Western Province is adjacent to the Torres Strait in Australia, and therefore, the PNG prioritisation and risk assessment provided more information that the Torres Strait and consequently, Cape York Peninsula are considered a high-risk region for entry of rabies to Australia (Figure 2).

Accurate data are important for accurate model predictions. Most rabies risk assessments investigate illegal pathways which makes accurate data inherently difficult to obtain. However, sensitivity analyses highlighted that knowledge of prevalence of rabies at origin is needed to accurately determine the risk of an incursion. In the context of rabies, this is a persistent knowledge gap worldwide, which not only influences the accuracy of risk assessments, but also rabies surveillance and control. Rabies is not detectable prior to development of clinical signs in animals and therefore, the prevalence of rabies in dog populations cannot be accurately determined. Given the potentially long latent period, it is possible for rabies to remain endemic at low prevalence in dog populations.

The results of risk analyses are specific to one pathway, and therefore, do not represent the total risk of incursion in Australia. The total risk of an incursion of rabies is additive across many pathways, for each mode of transport and each location. Additionally, risk distribution changes with a range of factors that have political, social and economic drivers. This is particularly important in the context of PNG where the volume of traffic on a pathway most influenced risk, and where ongoing land-use changes associated with logging and the palm oil industry influence human migration across the border from Papua Province, Indonesia. Although Western Province in PNG was identified as the highest-risk region (mainly hunters and logging), this might change in the near future.

Overall, unless prioritisation and risk assessments are conducted regularly—either routinely or in response to information from horizon or environmental scanning—investigation of a single pathway or even a group of pathways only gives a snapshot of risk at the time. Therefore, their main benefit lies in sensitivity analyses, which are more likely to be generalisable across pathways. To prevent an incursion in Australia, the most influential strategy would be to reduce prevalence, or eliminate canine rabies at origin—highlighting both the importance of pre-border biosecurity, and global strategies such as ZeroBy30.

## 5. Disease Modelling: Mitigating Expected Impacts of RABV in Australia

The objectives of disease modelling are to understand disease spread mechanisms and to make predictions about disease impacts. The latter can be done by simulating spread in a specific population under various circumstances, and in the context of this review we focus on disease spread modelling as a component of disease preparedness. Impacts can be described in terms of disease incidence and distribution (demographics affected, as well as spatial and temporal patterns), duration of outbreaks, and the economics of control strategies. Such information can be useful for surveillance or response purposes; for example, identifying the highest risk groups in a population for targeted surveillance, and identifying optimal control strategies. Disease modelling is, therefore, a natural progression in a program for disease preparedness following investigation of incursion risks using risk assessment. It is also similar to risk assessment: although sensitivity analysis provides insights about influential parameters, valid predictions (and thus the greatest benefit) depend on accurate data to parameterise the model. In the case of disease modelling, transmission parameters are critical. Therefore, accurate data about the population of interest—such as their demographics, dynamics, movement and contacts—are needed. Following prioritisation and risk assessment of potential routes of incursion, the regions of interest for RABV spread modelling in the program presented here were Western Province, PNG, and the Torres Strait and the NPA, Australia (Figure 2). Consequently, research initially focused on collection of demographic and contact data of dog populations in these regions. 

In Australia, dog populations of interest are free-roaming, owned, domestic dogs in remote communities in the Torres Strait and the NPA, and the free-living dingo population in the NPA. Methods to investigate their demographics, patterns of movement and contacts included systematic review of the literature [72], surveys of dog owners in communities [73], GPS telemetry data from individual collars on domestic dogs [74,75,76], and movement and heat- and motion-triggered camera traps placed around communities and in the surrounding bushland [77,78]. These data were collected over a period of two years. Demographics, population dynamics, roaming ranges and contact frequencies were estimated for domestic dogs, peak activity periods and spatiotemporal overlap were estimated for domestic dogs and dingoes, and home-range size and density were estimated for dingoes. Thus, population parameters were available for Australian models of RABV spread in domestic dogs in the Torres Strait and NPA communities, and dingoes in the NPA. Importantly, thematic maps of the distribution of domestic dogs (and the extent of their roaming), dingoes and the potential sites of interaction between these populations were developed during an intense phase of field research [76,77,78,79,80,81].

Agent-based, stochastic, rabies spread simulation models were subsequently developed for domestic dogs in Torres Strait island communities [82], NPA communities [83,84] and the dingo population in the NPA [85]. The key advantage of agent-based models compared to previous rabies models developed for Australian conditions [86,87], is that population mixing—and thus contacts between infectious and susceptible individuals—are heterogeneous. This was particularly important in the case of the domestic dog populations. In the NPA, different roaming-types were identified which influenced the spatial overlap, and thus the potential contacts of dogs; in the Torres Strait, roaming dogs formed small-world networks in which there were small clusters of strongly-connected dogs (in terms of time spent with each other), with connections (albeit weaker) between most groups. This heterogeneity of contacts influenced the predicted impacts of vaccination strategies.

In the context of outbreak response, the NPA model predicted that pre-emptive vaccination strategies that had covered 10–50% of the population and targeted far-roaming dogs rather than dogs that stayed at home reduced outbreak duration and size, especially if the response following outbreak detection aimed to then vaccinate 70% of all dogs. The network-based model of the smaller populations of dogs in the Torres Strait further accounted for heterogeneity of contacts by incorporating rabies-induced behavioural changes. In the context of prevention of disease emergence, it was predicted that maintenance of vaccination of >80% of the population (rather than 70%) was required to prevent emergence of rabies in such populations. 

These results highlight a feature of models in that outcomes are influenced by the structure of the model even when the same disease is modelled. As was illustrated in this program, the structure of a model is driven by the model’s purpose, and consequently, interpretation of findings should be made only in the context of this purpose. Implementation of model findings in policy is then further determined by social, economic, political and logistical factors that act both locally and more globally. Given the geographic interface that the Torres Strait islands naturally create between PNG and the Australian mainland, and the low likelihood of timely detection of rabies in PNG, we focused on prevention of emergence when modelling rabies in the small populations of dogs in this region and recommend that all domestic dogs in the Torres Strait should be pre-emptively vaccinated. In the case of the NPA, the dog population is larger, but less stable (i.e., dogs are brought in by residents and holiday makers for hunting). This makes vaccination logistically more difficult; however, compared to PNG, detection of an outbreak is likely to be more timely given the greater surveillance resources [88]. Recommendations to pre-emptively vaccinate a smaller proportion of the owned, domestic dog population, particularly dogs that roam, and respond with vaccination in the face of an outbreak, is supported by modelling. 

Rabies spread modelling in the dingo population of the NPA region predicted that a median of 14% of the population (22 dingoes, 95% range 2–101 dingoes) would be infected, with a median rate of spread of 0.52 km/week for 191 days. Predicted outbreaks were larger in scenarios in which an incursion was introduced during the dry season or close to communities. This suggests that although model outputs indicated that the dingo population would not support sustained propagation of rabies, the risk to domestic dogs and people in local Indigenous communities needs to be considered, for example in further investigation of potential control strategies such as ring vaccination around communities. It also highlights that more needs to be known about the nature of the interface between domestic roaming dogs and dingoes [78]. Sensitivity analyses indicated that home range size and the duration of infection most influenced model outputs. Therefore, the model could be further refined by improving data about dingo home range in the NPA by GPS tracking. 

**Figure 3:**
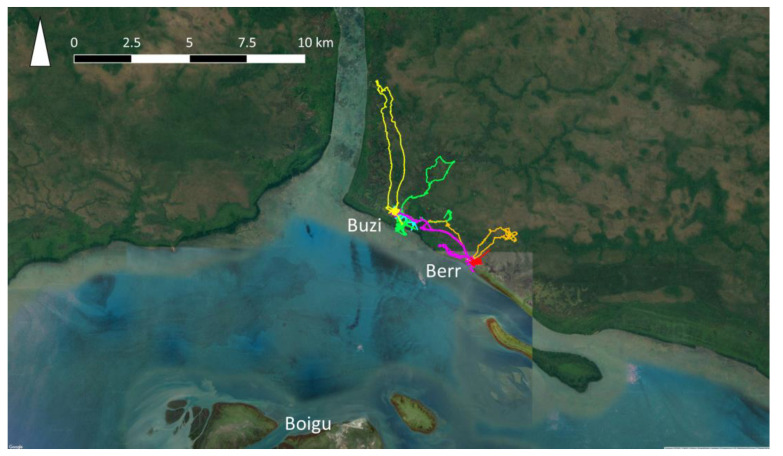
GPS trajectories from the collars of six owned, domestic dogs (each represented by a different colour) in Buzi and Berr villages, Western Province, Papua New Guinea.

Data on dog populations in Western Province, PNG were more difficult to collect due to the logistic constraints of travel in this region. In 2018, GPS telemetry data were collected from 60 domestic dogs in treaty villages Kadawa, Buzi and Berr, in Western Province, PNG (Figure 3), to estimate home ranges using utilisation distributions [75]. Buzi and Berr dogs roamed over significantly larger HRs than dogs in Kadawa. This might have been due to hunting dogs being collared in Buzi and Berr, and not in Kadawa. It was also clear that dogs travelled between Buzi and Berr, thus extending their home ranges. These data highlighted that there are significant differences in the movement and contact patterns of dogs between villages that could influence model outcomes. In addition, data were incomplete regarding dog population size, demographics and dynamics, as well as the frequency of dog travel between villages. Given the number of villages on the coast of Western Province, these gaps in the data and suspected variation in dog activity between villages, were considered too great for meaningful rabies modelling in Western Province because outputs could be misleading. Data on the movement of dogs between villages on foot or by boat would be of particular value, because information about the expected speed of rabies spread between villages is critical for disease preparedness in this remote region due to the lack of surveillance. Once an incursion of rabies is detected, prediction of potential spread would be required to inform the size of control areas for movement restrictions, vaccination and quarantine strategies. Therefore, this is a large gap in preparedness for the Oceanic region. 

## 6. Discussion

The research program presented here has produced many outcomes. In terms of the emergence of canine rabies in the region, it has contributed to policy, response planning and public awareness. For example, the response policy for an incursion of rabies in Australia (AUSVETPLAN) is updated regularly, and the forthcoming update (version 4) will incorporate some findings from the research program such as risk pathways and options for control. In addition, gaps have been identified. These include the need for methods development in remote regions such as PNG, and characterisation of the wild dog–domestic dog interface. These gaps are a challenge particularly for disease spread modelling. Likewise, the use of participatory methods to source information to parameterise risk analysis models within this research program offers an approach to overcoming this data input gap. Some of these methods have been applied successfully in both the Torres Strait and Papua New Guinea [70,88,89,90]. These inputs are critical to produce reliable outputs from models, and hence to inform policy and guide decision making.

The framework in which this program was conducted only considers rabies in the context of epidemiologic risk (including impact) and the requirements of preparedness. It can be extended by using methods from fields such as anthropology and social science; for example, to determine feasibility and acceptability of strategies. This is particularly important during the translation of recommendations based on epidemiologic outputs into effective policy. Some research was conducted in the area of social norms and behaviours, and how these might influence the recognition of an incursion, and the response that is mounted. For example, in studies conducted in areas identified as high risk of a rabies incursion via risk assessment, dogs and dingoes were found to have great cultural importance, but this varies within and between the communities and has implications for rabies response. Dogs are not equals or deities; nor is harm to them inconsequential. There are also different concepts of ‘ownership’ and dog care practice. During the first phase of a rabies response, seizure and destruction of infected and exposed dogs would be tolerated if prior permission is sought and received, and all animals are treated with respect based on their role as a family member and part of the wider community. A vaccination program would be welcomed, but movement restrictions would be burdensome if not impossible due to poor quality fencing and food security concerns. The culling of healthy dingo populations would be problematic in all settings due to their high cultural importance. The capacity of communities to contribute or adapt to the rabies response policy is variable and limited by the lack of infrastructure and services, lack of appropriate information, dominant cultural norms (among people and dogs) and food security concerns. This research found that communities need to be aware of risks and consequences prior to an outbreak [90]. Social research needs to be incorporated into emerging disease policy, planning and response, but its use remains limited, perhaps because scarce resources are prioritised to determine technical requirements such as the number of vaccines required, rather than how best to encourage people to vaccinate their dogs. These are generic issues, relating to not just an incursion of rabies.

The value of continued environmental and horizon scanning to develop an understanding of drivers of EIDs such as rabies in the Southeast Asian region cannot be underestimated. Despite numerous regional initiatives—including harmonisation of regulatory, strategy and standard guidelines; vaccination and PEP; dog population management; capacity building; and stakeholder engagement—the rabies situation in Southeast Asia has deteriorated over the past three decades, as rabies has spread to areas that were previously free. There has been a resurgence of rabies in Thailand, northern Vietnam and West Kalimantan. The northern part of West Malaysia reported a rabies incursion in 2015, and Sarawak in East Malaysia in 2017. In January 2019, an outbreak in Sumbawa, Nusa Tenggara Barat, Indonesia was reported. Within the context of ongoing rabies spread within the Southeast Asian region, the goal of regional rabies control is challenging. Key technical challenges include inaccurate estimates of the size of dog populations, inconsistent dog population management and low vaccination coverage. Understanding the drivers of rabies spread for strategic prevention of incursions would be beneficial.

Surveillance to detect rabies in dogs before humans are affected also remains a challenge. In July 2017, three children in Serian District in the east Malaysia state of Sarawak were admitted to hospital with clinical rabies. East Malaysia (the states of Sarawak and Sabah) was historically free of rabies. In the adjacent Indonesian provinces of Kalimantan, canine rabies had progressively spread since an incursion in East Kalimantan province, reported in 1974. The first reports of rabies from West Kalimantan province, which shares a border with Sarawak, were in 2005 from Ketapang District, in the south of the province. This outbreak was short lived. Rabies then re-emerged in 2014 in Ketapang and Melawi Districts, which both share a border with Central Kalimantan province, the assumed source of the incursion. Between 2014 and 2017, rabies apparently spread throughout West Kalimantan province. By 2018, all districts except the provincial capital had been affected, with increasing reports of dog bites and human rabies cases. During 2018, rabies spread throughout many districts of Sarawak.

Apparently, the incursion of rabies into Sarawak was not anticipated. In this situation, environmental and horizon scanning might have identified such a future scenario. However, in 2019 an investigation of drivers of rabies spread and maintenance was undertaken [91,92]. Information was collected via field observations in West Kalimantan, a range of online data and discussion lists, and both local and regional expert opinion, and trends were identified. Although conducted in retrospect, this example illustrates how environmental scanning might be implemented to anticipate a rabies incursion scenario.

The border between West Kalimantan and Sarawak is 857 km in length. There are three official border posts (Aruk, Entikong and Nanga Badau), but there are many unofficial crossing points. For example, in one district with an official border post, there are at least 54 other unofficial crossing points. People generally cross the border at unofficial points to visit family, and for trade and work. Crossing for medical treatment usually occurs at the official border points. Dayak people (traditional inhabitants) regularly cross the border in both directions for trade. Most border crossing is on foot and occasionally motorbike. The border is indistinct (for example, not defined by a river) and some households even straddle the border.

There are estimated to be 200,000 dogs in West Kalimantan, probably much higher than in adjoining Sarawak. This can be partly explained by the use of dogs (interchangeably) for hunting, protecting homes and crops, as ‘special’ dogs (pets and dog guardians) and for consumption. Many villages in West Kalimantan are predominantly Christian, so dogs are more valued in these villages. Other notable features of West Kalimantan society are harvest festivals (Gawai) and a substantial indigenous population (the Dayak).

The scanning of several information streams might have led to scenarios in which an incursion of rabies from West Kalimantan to Sarawak were a feature. These include:

- An increase in **demand for hunting dogs**. In this area, dogs are used to hunt pigs, and it appears that most of the meat is consumed within villages. However, demographic changes in the region (for example, increased disposable income, or changes in the cultural mix of the population) in which there is an increased demand for pig meat and therefore an increased price paid, could be a driver of a larger hunting dog population and therefore increased rabies spread.
- An increase in **demand for dogs to guard crops**. Dogs are used within the region to guard crops from wildlife, such as wild pigs and monkeys. As above, an increase in demand for crops due to immigration or socioeconomic changes could drive an increase demand for guard dogs. In 2019, a rabies incursion in Sumbawa, Indonesia was reported. Initial investigations suggested that this incursion in the previously rabies-free island was due to the movement of dogs from either Bali or Sulawesi, Indonesia, for the purposes of guarding corn crops from wild monkeys.
- **Ethnic, cultural and religious trends**. In the district studied, it appeared that in rural areas rabies is more likely in villages that are predominantly Christian, versus those that are predominantly Muslim. It is also likely associated with indigenous Dayak populations. The association between ethnic, cultural and religious affiliations and dog ownership and movement have been previously described [93].
- **Gifting of puppies** has been linked to the spread of rabies to previously free areas, such as Tanibar, Maluku Province, Indonesia. It appears that gifting puppies is a traditional practice in the region. However, increased disposable income and an increase in pet shops or other pet traders might increase the risk of rabies spread.
- **Dog meat consumption**. Most dogs appear to eventually be consumed when their other uses are exhausted. This is a local activity and likely not a potential driver of regional rabies spread. However, the most likely reason for long-distance movement is supply of dogs for consumption (especially during the harvest festivals between January and June) if dogs for consumption are scarce in the local area. Gawai (harvest festivals) are held in every village between January and July. The time varies between villages due to the harvest time. There is also a district Gawai. Dogs are consumed during these festivals. This system of festivals appears to be relatively constant (and perhaps even decreasing in popularity). Changes that promote traditional festivals—such as an increase in tourism—could create a scenario of rabies spread via dog movements for consumption.
- **Regional road improvements**. Major roads throughout the region have been sealed within the past 5 years. Better roads enable increased trade and travel. If dogs are taken on such trips, then the risk of rabies spread can be expected to increase in a future scenario.
- **Oil palm plantations**. The increasing number of oil palm plantations in the region has provided opportunities for work and trade. The oil palm plantations are numerous on the Malaysian side of the border and have increased unofficial, local border travel. Indonesians work on Malaysian owned plantations (forest on the Indonesian side, oil palm on the Malaysian side) and also trade (in Malaysian currency). Dogs are used on oil palm plantations for protection, although details on the roles played by dogs on oil palm plantations are not readily available.

Information on some of the above are likely available; for others, sourcing information might be challenging. For example, information on roads tends to be readily available in most countries and regions via satellite imagery and GIS. Data include the total length of roads, the number and length of major and sealed roads, and the connectivity of the road network. In some cases, traffic volumes (including at the number of passengers and amount of freight) are available. Therefore, changes in the road infrastructure and network can be scanned to determine if risk of spread of a contagious disease is increasing or decreasing. In contrast, in the border area of West Kalimantan and Sarawak, information on oil plantations is less readily available—although crude measures of the changes in area under management is possible via satellite imagery. Changes in economic activity and disposable income are often available. However, more proximal changes in drivers of the spread of a disease such as rabies—the number and cost of dogs—often are not. Here, an alternative might be to scan the prices of dogs for sale at markets or in pet shops. The initial incursion of rabies in West Kalimantan was in the southern district of Ketapang. This district is known for its restaurants which serve dog meat. Scanning the prices paid in these restaurants would provide a data source to inform disease incursion scenarios. An issue when considering information sources and their ease of access is whether the activity is legal or not.

Overall, a systematic, integrated research program for rabies preparedness in the Oceanic region was feasible using our previously described framework. In circumstances in which data were limited, some outputs were limited (for example, rabies spread modelling in PNG). Whilst this is a barrier to any research, using the framework clearly identified the gaps and the ongoing requirements for preparedness. The framework is iterative at all stages, which serves as a reminder that outputs need to be updated as inputs evolve. The situation in West Kalimantan, Sarawak and Sumbawa illustrate not only the need for continuous assessment of data streams, but also continuous updating of research outputs to re-evaluate priorities, risks and potential impacts of rabies spread.

Surveillance for a disease such as rabies in a resource-limited setting is challenging to maintain over time [94], and routine analysis remains problematic [95]. The former can be addressed in scanning programs, such that secondary data that are already available are utilised. The specificity of scanning programs is less than a bespoke rabies surveillance program, and it also has a longer time-scale in terms of preparedness and response. This has advantages: multiple such EID preparedness frameworks can be initiated with scanning data, making use of these data efficient through enabling a broader One Health agenda for strategic and tactical planning, and enhancement of support for human, animal and environmental health.

Most programs in human and animal health tend to be disease specific and consequently, a systematic framework such as that described in this paper only vertically integrates investigations to develop prevention, detection and response strategies. Focusing on response to one disease can be detrimental to the control of others, and the COVID-19 pandemic has highlighted that horizontal integration across diseases and human and animal health sectors is important and should not be overlooked. For example, a recent modelling study suggests that reduced dog vaccination and rabies surveillance due to COVID-19 control measures in Arequipa, Peru will result in increased human risk due to rabies [96]. In a broader context, it has also been suggested that frameworks for investigation and management of malaria could be extended horizontally, and used as surveillance for non-specific EID surveillance by investigating all reports of fever in people [97].

Finally, given the goal of ending human deaths from dog-mediated rabies by 2030 (“ZeroBy30”), the need to prevent the spread of rabies to areas historically free of disease—and also the spread to areas where dog-mediated rabies has been eliminated—will become increasingly important in the latter phases of the campaign to achieve this goal. ZeroBy30 will also require further development of appropriate surveillance methods to demonstrate freedom from rabies and timely detection of incursions.

## 7. Conclusions

Although a range of tools have been developed which can be applied to address EID event detection, preparedness and response, a systematic approach that integrates these tools has rarely been applied. Considering the number and characteristics of these tools, it can be daunting to begin such a process: threats are dynamic and evolving, and data needs can be overwhelming. However, the application of foresighting techniques can begin simply by monitoring those factors known to promote EID events, such as changes in trade, animal management and transportation; such information is collected through environmental and horizon scanning. This can be integrated with passive surveillance data to provide intelligence on threats. Simple, qualitative risk assessments can also add value. As these activities mature and outputs are integrated, gaps become apparent and additional data collection can be planned to parameterise more complex risk assessment and disease spread models, as required. As part of a dynamic system, outputs from such models likely will direct further research to fill data gaps.

The information generated needs to be evaluated regularly in terms of the goal of such a framework, i.e., reduce the probability of an EID event and, should an EID event occur, detect it early and mitigate its impact. In addition, the scale at which activities need to take place (such as region, country or community), and how policies generated are implemented (including anthropology, behavioural and social sciences), need careful consideration for effective outcomes.

## Data Availability

Not applicable.
